# Disruption of Circadian Rhythms: A Crucial Factor in the Etiology of Depression

**DOI:** 10.1155/2011/839743

**Published:** 2011-08-08

**Authors:** Roberto Salgado-Delgado, Araceli Tapia Osorio, Nadia Saderi, Carolina Escobar

**Affiliations:** ^1^Departamento de Biología Celular y Fisiología, Instituto de Investigaciones Biomédicas, Universidad Nacional Autónoma de México, 04306 México, DF, Mexico; ^2^Departamento de Anatomía, Facultad de Medicina, Universidad Nacional Autónoma de México, 04306 México, DF, Mexico

## Abstract

Circadian factors might play a crucial role in the etiology of depression. It has been demonstrated that the disruption of circadian rhythms by lighting conditions and lifestyle predisposes individuals to a wide range of mood disorders, including impulsivity, mania and depression. Also, associated with depression, there is the impairment of circadian rhythmicity of behavioral, endocrine, and metabolic functions. Inspite of this close relationship between both processes, the complex relationship between the biological clock and the incidence of depressive symptoms is far from being understood. The efficiency and the timing of treatments based on chronotherapy (e.g., light treatment, sleep deprivation, and scheduled medication) indicate that the circadian system is an essential target in the therapy of depression. The aim of the present review is to analyze the biological and clinical data that link depression with the disruption of circadian rhythms, emphasizing the contribution of circadian desynchrony. Therefore, we examine the conditions that may lead to circadian disruption of physiology and behavior as described in depressive states, and, according to this approach, we discuss therapeutic strategies aimed at treating the circadian system and depression.

*Tell me; what is the day or night to one who is mired in grief?* —William Blake. 

*Life is full of joy and sadness. But when sadness lasts too long or interferes with the capacity for developing daily chores, it is possible to have a common and serious disorder: depression.*

## 1. Introduction

Depression affects mood, mental and physical condition, and behavioral performance; it is expected that by 2020 it will be the second cause of incapacity in adults (http://www.who.int/mental_health/management/depression/definition/en/). Approximately each year, 20 million people, one of ten adults, suffer from depression, and approximately 60% of these individuals do not receive the needed help, even though treatment diminishes symptoms in more than 80% of the cases.

According to DSM-IV, the major depressive disorder is generally classified in unipolar, bipolar, or seasonal subtypes. Depression is defined as an affective disturbance with chronic sad mood state (dysthymia) and with a loss of interest in new or previously pleasant activities (anhedonia). 

It is accompanied with guilt and feelings of uselessness, and in some cases, suicidal ideation [3]. Behavioral and cognitive difficulties, such as memory or attention problems, are observed in depressive patients [4]. Sleep perturbations such as insomnia or excessive drowsiness, as well as feeding problems with excessive or null appetite are characteristics of this disorder [5].

**Figure 1:**
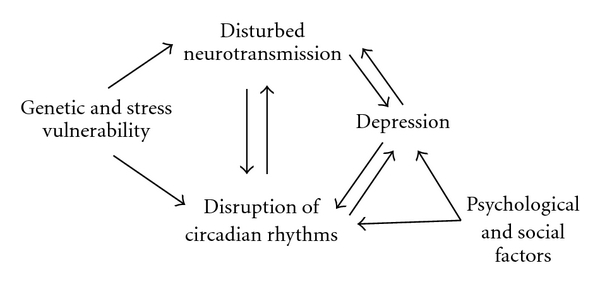
Potential model of depression. The reciprocal relationship between the disruption of circadian rhythms and behavioral disorders may be either cause or effect of a depressed affective state, and it is influenced by genetic, neurochemical, and neuroendocrine factors [1].

The etiology of depression is complex; it may have a genetic, physiological or hormonal origin or be triggered by stressful conditions and/or psychological and social factors [1] (Figure 1). In addition, studies in chronobiology indicate that the disruption of circadian rhythms also contribute to the onset of depression [6]. Two factors leading to circadian disruption are the increase of nocturnal activity which is accompanied by a decrease in sleeping time and the extended exposure to artificial light at night.

## 2. The Circadian System and the Relevance of Biological Rhythms

**Figure 2:**
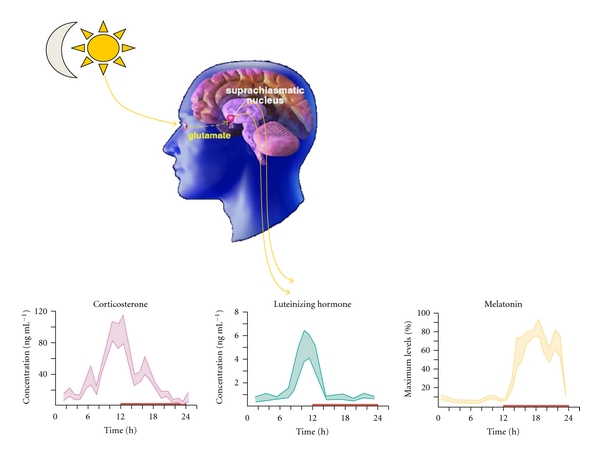
Targets of the SCN. The temporal signal transmitted by the suprachiasmatic nucleus, the biological clock, is translated into a rhythmic hormonal pattern or signals provided by the autonomic system. This relationship provides information to the body that adjusts peripheral organs to the light-dark cycle. The bottom panel shows the circadian rhythm in blood levels of corticosterone, luteinizing hormone, and melatonin for rodents modified from [2].

Biological rhythms are related to geological cycles like night and day, summer and winter, resulting from earth's rotation and translation, respectively. These geographic events impose adaptation processes to all living organisms, which have to adjust their physiology to periodic environment fluctuations. Thus, organisms present rhythms that are synchronized to daily and annual cycles, which are called circadian and circannual rhythms, respectively. The mechanisms generating temporal events in the organism were ignored until the 70's, when researchers discovered that the lesion of a small hypothalamic site above the optical chiasm, named the suprachiasmatic nucleus (SCN), led to loss of circadian rhythms [7, 8]. This finding was the first evidence that in the brain there is a structure that functions as a master clock which gives a temporal order to internal processes in the organism [2] (Figure 2).

**Figure 3:**
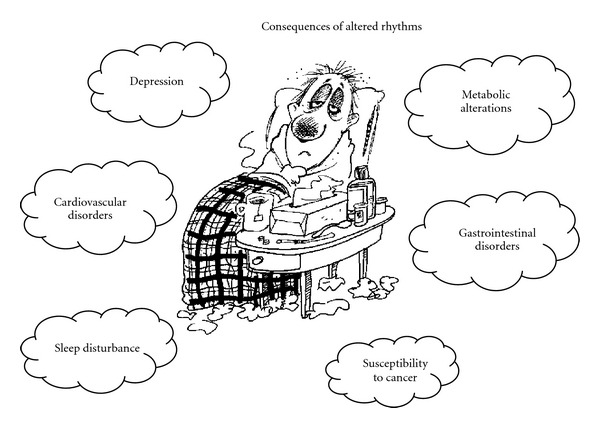
Schematic representation of health problems associated with internal desynchronization. The figure shows health problems that result from physiological changes in biological rhythms.

The SCN coordinates daily sleep-wake cycles, metabolic processes, hormonal release, and in general the temporal order of all the body physiology. It also coordinates temporal oscillations of cells and organs, coupling the organs and systems to function in harmony [9]. The alteration of this temporal order causes sleep disturbances, irritability, lack of attention, gastrointestinal, or heart diseases as well as tendency to develop cancer, however, the mechanism by which circadian disruption leads to disease requires further studies [10–14]. It is well known that altered activity and sleep patterns can lead to desynchrony, as observed in shift and night workers and in individuals exposed to frequent transmeridional trips (jet lag) [15, 16]. Modern lifestyle is also turning to be a relevant factor promoting desynchrony, especially in individuals staying for long hours awake during the night and with disturbed sleep patterns [17, 18] (Figure 3).

## 3. Light Pollution Repercussion on Health

In nature, light has a powerful influence in living organisms. It is known that light stimulates special retinal cells which project to the SCN, where they release glutamate as neurotransmitter. In this way, the SCN is continuously informed about environmental light/dark cycles as the main Zeitgeber or synchronizing factor for coupling all functions and behavior to the external day/night alternation [19]. 

Due to the relevance of the day/night cycle for the body's temporal order, alterations of the exposure to light may alter the internal circadian rhythmicity. In humans, which are diurnal organisms, light plays a relevant role on life because all the activities are carried out during the day. In addition, to have a sufficient and satisfactory sleep during the night is crucial for physiology and behavior [20]. In fact, during sleep a series of relevant physiological events take place including, cellular repair and mental recovery, necessary for health and survival of all individuals. Physiological processes also depend on sleep, serotonin, which is a neurotransmitter that modulates the mood state, and diminishes its levels during the night, while melatonin, a hormone that is synthesized in the pineal, is released during the night inducing sleep and stimulating DNA's repair [21–23]. Thus, these neuroendocrine rhythms, together with a large number of other physiological processes, are programmed to occur at a specific moments of the circadian cycle, to guarantee an adequate rest and prepare the body for diurnal activities [2].

Not so long ago, only sun light or an intense artificial light (between 7.000 to 13.000 lux; similar to 12 hr generated) was thought to readjust the biological clock and, therefore, to modify circadian rhythms [24]. However, recent studies point out that the human biological clock is much more sensitive to light changes, and the disturbance of circadian rhythms also results from low-intensity light exposure [24–26]. In healthy volunteers different light intensities, ranging from 0.03 to 9.500 lux, during the night cause in short term an important impairment of temperature and hormonal rhythms [27]. Disruptions of circadian rhythms were similar in both conditions of dim or bright light [24]. Such new evidence indicates that humans react to artificial light at low or high intensity (around 180 lux), which means that the light intensity used for illuminating house interiors and job areas are sufficient to alter the biological clock and circadian rhythms. The consequence of a shifted clock is manifested with insomnia, nonsufficient rest, melatonin inhibition, and hormonal impairment [17, 28]. 

The relationship between exposition to light at night and the onset of a number of serious pathologies are not yet clear, because it is a relatively new phenomenon for human society. At the present time, city inhabitants are exposed, during the first hours of night, to light levels around 1.000 lux [29–31]. 

**Figure 4:**
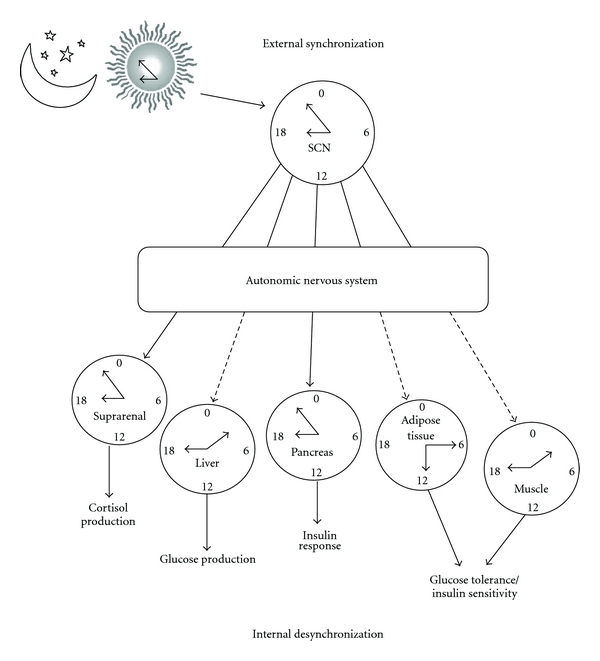
Schematic representation of a state of internal desynchronization. The biological clock (SCN) is synchronized with the dark-light cycle (external synchronization), and, in turn, the SCN synchronizes peripheral oscillators (internal synchronization). However, some oscillators may lose their synchronization with the SCN (dotted lines) due to alterations in the rhythmic output from the clock to the rest of the body or due to the interference given by nonphotic entraining stimuli, especially disturbed activity and feeding schedules. This uncoupling between the central clock and the peripheral oscillators is known as internal desynchronization, which results in physiological responses at the wrong time according to external requirements, which then leads to disease.

Studies with animal models are providing an alternative for determining the consequence of long-term light at night exposition and indicate deleterious effects on behavior and physiology, as well as on molecular mechanisms of the clock [32–34]. Experimental studies with rats clearly demonstrated that repetitive exposure to dim light (50 to 300 lux) during the night for a relative short period of time (5 hrs average) have similar effects on circadian rhythms as bright light [35, 36]. This exposure to artificial light, which has an intensity to the one generated by a 60 watt bulb, for short periods of time during the night induces an important shift in the biological clock advance. In rodents exposure to constant light leads to irritability, anxiety like and depressive-like behaviors and altered deficits in learning and memory [37–41], inhibition of melatonin secretion, accelerated aging and tumorogenesis, increase in visceral adiposity, propensity to obesity, and cardiovascular function [42–44]. All this together conforms that the disruption of circadian rhythms consequent to an a overexposure to the artificial light may lead to behavioral and physiological dysfunction [45] (Figure 4). 

## 4. Modern Lifestyle Disrupts Circadian Rhythms

Besides photic signals, there are also nonphotic entraining stimuli influencing the biological clock as time indicators (Zeitgebers), such as those given by feeding schedules and physical activity [46–48]. Such stimuli can also give temporal signals to the SCN, although they are weak as compared with light inputs [49]. Although they do not override the light/dark cycle for the adjustment of the SCN, nonphotic stimuli, especially food, contribute as potent synchronizers of cells and organs in the periphery, driving them out of phase from the signals transmitted by the SCN, thus resulting in internal desynchrony [50–52] (Figure 4).

**Figure 5:**
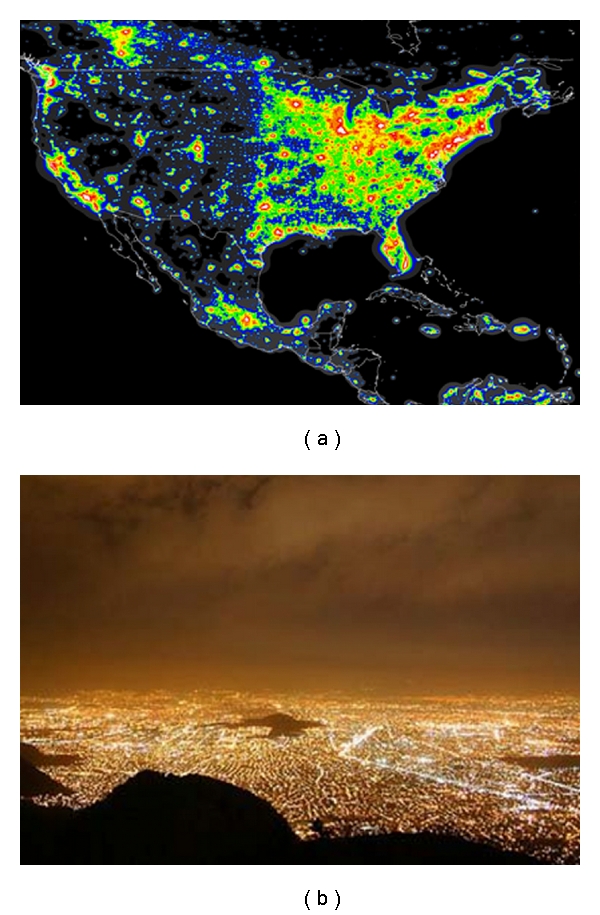
(a) a map of U.S. and Mexico at night, seen from a satellite: red and yellow colors represent up to 200 times the natural level of brightness. (b) an aerial view of Mexico City: note the enormous light pollution (http://www.lightpollution.it/).

In humans, social activity represents the second most important Zeitgeber influencing the biological clock, while the alternation of light/dark cycle is still the most important entraining signal. The development of modern technology has promoted a relative independency of social and work activities from the environmental light/dark cycle. Thus, artificial light allows night activities, including night work, possibly affecting the function of the biological clock of people exposed to frequent nocturnal activity [53]. Some examples of modern-life habits that alter our biological rhythms are given by the long flights across the continents (jet-lag), shift work, night work, and so forth, [28, 54–56]. Animal models simulating similar conditions as night work have confirmed the deleterious effects of activity during the resting hours and circadian desynchrony [57–60]. Even more, the proportion of individuals that stay awake for long intervals in the night, engaged in leisure activities, has importantly increased and has become an important health problem [61]. Remaining awake in the night promotes physical activity and arousal, in addition, by being awake individuals tend to eat at hours when the biological clock indicates sleep time [62]. Considering the strong entraining power of feeding schedules on cells and organs of the periphery, such disturbed activity schedules and constant sleep deprivation lead to a disruption of the internal temporal order and lead to anxiety, depression, and altered behavioral performance [63, 64]; this emphasizes the importance of synchronicity for mental health. Because young people and new generation are shifting their temporal patterns towards the night, the impact of night activities on behavioral performance and mood requires more research and is becoming a topic of high priority (Figure 5).

## 5. Depression and Circadian Rhythms

Circadian variations of behavioral, physiological, and metabolic factors are common in patients with unipolar and bipolar disorders [65, 66]. The most evident variation is observed in mood, because commonly depressed individuals show an improvement of their mood state in the evening. Patients report feeling better in the afternoon than in the morning which generally is inverted in bipolar depression [5]; likewise, the sleep/wake rhythm is affected by insomnia at night and hypersomnia (sleepiness) during the day, with a consequent feeling of constant tiredness [67, 68]. It is possible that the disruption of the sleep/wake cycle could be the main cause of alterations of circadian rhythms in body temperature, release of hormones, and metabolites related to the sleep time [63, 69–71]. In depressive patients, nocturnal melatonin release is often diminished, and this data may be related to sleep disturbances that depressive patients report [72, 73]. Approximately, 90% of depressive patients complain about the low quality of their sleep. It is not surprising then that brain regions involved in depression are also implicated in the regulation of the sleep/wake cycle. One possible link for the narrow relationship between sleep disorders and depression is the onset of and anxious state that affects depressive patients when they wake up in the morning [74]. Actually, the therapy with antidepressant drugs improves the quality of sleep, although some of them display collateral effects that aggravate insomnia and cause sedation and sleepiness during the daytime [75].

Depressive individuals also display increased levels of plasmatic cortisol, a hormone that is associated with stressful conditions. This constant hypercortisolemia has been reported to lead to an anhedonic behavior and metabolic alterations [6, 66]. In depressive patients, the circadian rhythm of cortisol is disturbed, indicating that an alteration of the internal temporal order *per se* may trigger the depressive symptomatology. 

## 6. The Winter Brings Lack of Light and Changes the Mood

In winter, when day is shorter and night is longer than in spring and summer, depression is developed as a consequence of a diminished sunlight exposure. Winter depression is characterized by the usual depressive anhedonic mood symptoms, but the vegetative symptoms are atypical hypersomnia, increased appetite (particularly for carbohydrates), and weight. This transient mood disorder has been denominated seasonal affective disorder (SAD). This type of depression is characterized by a regular, annual episode of mayor depression during autumn and/or winter, and remission or episode of mania/hypomania during spring and summer [65]. SAD affects 2–5% of general population in temperate weathers and generally is more common in the countries where seasonal changes are more noticeable, being days shorter than night and luminosity very low during the day in the winter time.

In general, low luminosity in productive moments of the human life affects negatively mood state, especially if the individual remains in close spaces and without a sufficient illumination. Light deficiency can cause sleepiness in the day and insomnia at night, affecting people performances, and promoting fatigue during the day. Because of this light deficiency, several brain areas are not enough stimulated for releasing dopamine and serotonin, neurotransmitters that contribute to improved mood state [76]. Contrasting with major depression in SAD patients melatonin is released in higher proportions as described before [77–79].

## 7. Chronotherapeutical Strategies for the Treatment of Depression

Disruption of circadian rhythms is a relevant factor contributing to the pathogenesis of affective disorders, thus the recovery of correct internal-external circadian synchronization should be considered as a possible strategy for the therapy of depression. This implies that achieving a regular lifestyle may contribute to the stabilization of depressive patients, improving in this way the quality of their lives [74]. This strategy requires keeping strict schedules for sleep/wake cycles as well as the timing for meals.

It has been proposed that the change of light color and intensity, perhaps using bright white light, in the interior of the houses and in the work places during the day may ameliorate vision and the perception of the space around, preventing the onset of the depressive mood. Indeed, light therapy is a curative strategy often used in the treatment of individuals susceptible to recurrent depressive episodes in winter. This approach to depression postulates that a prolonged exposure to light in the early morning simulates the large photoperiod typical of spring and summer, preventing those symptoms associated to the shortening of the light time. Even more, this light therapy provided during the early morning also has shown to be efficient as antidepressant in nonseasonal depression (requiring usually longer treatment than SAD). Contrasting, the prevention of excessive light during the night has been hardly approached as a necessary measurement to prevent depression associated with sleep disturbances [80, 81].

Currently, the therapy of depression includes the pharmacological and different kinds of psychotherapy, cognitive behavioral therapy, and interpersonal psychotherapy. Given the number of secondary effects that antidepressant drugs may cause, it is common that some depressed individuals prefer to quit the prescribed therapy. For this reason, an increasing amount of clinical studies supports the use of chronotherapy as an alternative for the treatment of mood disorders. This proposal requires that the administration of antidepressant drugs should occur in the moment and dosage when the beneficial effects on the neurochemical systems are maximal and the collateral consequences are the less annoying for the patient [82, 83].

Other “chronotherapeutical” strategies include strategies that modify the biological clock, like, light or dark therapy, sleep deprivation, or a phase advance in the sleeping time. All of these approaches are directed to modify and adjust the biological clock to the correct phase [84–87] and ameliorate light exposure [88]. Also, some pharmacological products that affect directly the function of the biological clock have been proposed for a chronotherapeutical use. Melatonin and melatonin agonists have chronobiotic effects, which mean that they can readjust the circadian system. Administration of melatonin at the start of the night can be used to resynchronize the biological clock, by providing time signals and entraining sleep and thus reduces circadian disruption and probably some of the sufferings that occur in depression [89–91]. Seasonal affective disorders and mood disturbances caused by circadian malfunction are theoretically treatable by manipulating the circadian system. In major unipolar depressive disorder, melatonin alone has no antidepressant action, but novel melatoninergic compounds demonstrate antidepressant properties. Agomelatine, for example, is a new melatonin agonist and antagonist of the 5HT2 serotonin receptors, with antidepressant properties and capacity to regulate and ameliorate the quality of sleep [80, 92–94]. Furthermore, melatonin is a potent antioxidant and a promoter of the immune function, which are excellent side effects in the therapy of depression. In summary, antidepressants with intrinsic chronobiotic properties offer a novel approach to treatment of depression.

## 8. Conclusions

In the last years, a large amount of studies have focused on the understanding of the physiopathological mechanisms of depression. Evidence has pointed out that disturbances of mood may have their etiology in the perturbations of circadian rhythmicity, which are characterized by either delay or advance of the circadian phases, as well as by disorders in sleep and activity schedules.

The modern lifestyle promoting sleep deprivation, as well as light at night leads to circadian desynchrony. Disturbance of the circadian system leads to the loss of the internal temporal order, and to neurobiological and behavioral dysfunctions. Disturbances in the circadian sleep/wake cycle exacerbate a depressive state, due to altered patterns in the secretion of monoamines, such as serotonin, noradrenalin, and dopamine, regulating mood and involved in the pathophysiology of depression.

Chronotherapeutical strategies that reset or modify the biological clock may contribute to restore the internal synchrony and thus counteract psychological and physiological symptoms of depression. 

##  Disclosure

The authors have nothing to disclose.
